# Cellular Response against Oxidative Stress, a Novel Insight into Lupus Nephritis Pathogenesis

**DOI:** 10.3390/jpm11080693

**Published:** 2021-07-22

**Authors:** Corina Daniela Ene, Simona Roxana Georgescu, Mircea Tampa, Clara Matei, Cristina Iulia Mitran, Madalina Irina Mitran, Mircea Nicolae Penescu, Ilinca Nicolae

**Affiliations:** 1 Department of Nephrology, Davila Clinical Hospital of Nephrology, 010731 Bucharest, Romania; mirceapenescu4@gmail.com; 2 Department of Dermatology, Victor Babes Clinical Hospital of Tropical and Infectious Diseases, 030303 Bucharest, Romania; drnicolaei@yahoo.ro; 3 Department of Dermatology, Carol Davila University of Medicine and Pharmacy, 020021 Bucharest, Romania; 4 Department of Dermatology, Colentina Clinical Hospital, 020125 Bucharest, Romania; matei_clara@yahoo.com; 5 Department of Microbiology, Carol Davila University of Medicine and Pharmacy, 020021 Bucharest, Romania; cristina.iulia.mitran@gmail.com (C.I.M.); madalina.irina.mitran@gmail.com (M.I.M.); 6 Department of Nephrology, Carol Davila University of Medicine and Pharmacy, 020021 Bucharest, Romania

**Keywords:** systemic lupus erythematous, lupus nephritis, lipid peroxidation, DNA oxidation, oxidized proteins, carbohydrate oxidation, antioxidative stress strategies, biomarkers

## Abstract

The interaction of reactive oxygen species (ROS) with lipids, proteins, nucleic acids and hydrocarbonates promotes acute and chronic tissue damage, mediates immunomodulation and triggers autoimmunity in systemic lupus erythematous (SLE) patients. The aim of the study was to determine the pathophysiological mechanisms of the oxidative stress-related damage and molecular mechanisms to counteract oxidative stimuli in lupus nephritis. Our study included 38 SLE patients with lupus nephritis (LN group), 44 SLE patients without renal impairment (non-LN group) and 40 healthy volunteers as control group. In the present paper, we evaluated serum lipid peroxidation, DNA oxidation, oxidized proteins, carbohydrate oxidation, and endogenous protective systems. We detected defective DNA repair mechanisms via 8-oxoguanine-DNA-glycosylase (OGG1), the reduced regulatory effect of soluble receptor for advanced glycation end products (sRAGE) in the activation of AGE-RAGE axis, low levels of thiols, disulphide bonds formation and high nitrotyrosination in lupus nephritis. All these data help us to identify more molecular mechanisms to counteract oxidative stress in LN that could permit a more precise assessment of disease prognosis, as well as developing new therapeutic targets.

## 1. Introduction

Systemic lupus erythematosus (SLE) is a chronic autoimmune disease with complex pathogenesis, characterized by formation of autoantibodies against normal structures of the body such as skin, joints, blood elements, kidney, and central nervous system, with a heterogeneity of clinical manifestations. Skin lesions are frequent, such as symmetric malar erythema, photo sensibility, hyperkeratosis, ecchymosis, oral or mucosal ulcerations, alopecia. Proteinuria or nephrotic syndrome, changes in urinary sediment, increase in serum creatinine and arterial hypertension are clinical manifestations of lupus nephritis [1,2]. SLE—Autoimmune prototype disease—is characterized by abnormal responses of T and B immune cells with excessive synthesis of autoantibodies and immune circulant complexes [3] associated with nonimmune factors [4,5].

SLE clinical evolution is variable, being influenced by genetic factors, external factors, and human body resources. The pathogenic mechanism is promoted by the UV exposure of keratinocytes. Keratinocyte activation induces chemokines synthesis (CXCL5, CXCL8, CXCL20), production of adhesion molecules, apoptosis, keratinocyte photodistruction, release of nuclear and cytoplasmic antigens, and immune response initiation. These pathogenic events are processed by dermic and epidermic macrophages and presented to dermal naïve T lymphocytes. In SLE-related skin lesions, vacuolar and hydropic degeneration of keratinocytes and lymphocytic infiltration in papillary dermis could be found. Direct immunofluorescence reveals deposition in band of antibodies and complement to the dermo–epidermic junction. The cascade of autoantibodies induces the formation of circulant immune complexes, with preferential deposition in the synovial joint and glomeruli. The forms of LE limited to skin can evolve to SLE [6,7]. 

Oxidative stress in SLE was intensively studied during years. Reactive oxygen species (ROS) interaction with lipids, proteins, nucleic acids and hydro carbonates promotes acute and chronic tissue damage, mediates immunomodulation and trigger autoimmunity in SLE subjects [8,9,10,11]. Moreover, for their viability and correct functions, the cells develop endogenous strategies for suppression or modulating oxidative stress [3,12,13,14]. High levels of oxidative and nitrosative stress markers were determined in SLE patients with high disease activity. Still, oxidative stress influence immune and nonimmune cells on determining the disease phenotype in each subject [12]. An imbalance in oxidant/antioxidant equilibrium in autoimmune disease by endogenous or exogenous toxic factors exposure, by alteration of tissue damage response/repair mechanisms induces an aberrant activity of innate and adaptative immune response, high production of autoantibodies, multiple lesions of tissues and organs [4]. The bidirectional relation between oxidative stress and immune response, considered as part of autoimmune physiopathology, could change the paradigm of a disease characterized by perturbation of immune system and high production of autoantibodies [5,15,16,17].

A large diversity of lipoperoxides were detected in SLE in extracellular fluids and in blood. They influence disease expression by their effect on immune and non-immune cells [16,18]. Lipid peroxidation generates a variety of metabolites, the best known being saturated monoaldehydes; unsaturated aldehydes; dicarbonyls; malondialdehyde; 4-oxo-2-nonenal; hydroxydialdehydes (4-hydroxy-2-nonenal, 4-hydroxy-2-hexenal); oxidized phospholipids [8,19,20,21]. High levels of oxidative and nitrosative stress were detected in patients with active SLE, suggesting a link between lipoperoxidation and disease activity. Increased activity of malondialdehyde (MDA), 4-hydroxy-2-nonenal (HNE), MDA-protein adduct, HNE-protein adduct, superoxide dismutase (SOD), nitric oxide synthase (INOS), anti-MDA and anti-HNE antibodies were correlated in SLE patients with SLEDAI over 6 [9,16,21]. These lipoperoxides’ destructive effect could be limited by defense mechanisms of the human organism, such as lipoperoxides metabolization by oxide reductase (aldoceto-reductase, aldehyde-dehydrogenase, alcohol-dehydrogenase, glutathione-S-transferase) and cellular antioxidant defense mechanisms that include enzymes (SOD, chloramphenicol acetyltransferase—CAT, glutathione peroxidase—GPx, reductase—GR, S-transferases—GST, tioredoxin-reductase, hemoxigenase), non-enzymes (A, C, E vitamins), and carotenoids, flavonoids, glutathione and other antioxidant minerals [13,18]. Defense mechanism disruption was associated with clinical complications of SLE [10,11,16]. Isoprostan F2 (8-iso-PGF2) levels, a results of lipid peroxidation, was correlated with disease activity in SLE subjects. Moreover, high levels of MDA, F2-Isoprostan, nitric oxide and low levels of reduced glutathione were determined in patients with lupus nephritis [16].

In SLE, oxidative stress is involved in the formation of advanced glycation end products (AGEs) and advanced lipoperoxidation end products (ALEs), compounds with proinflammatory characteristics. AGEs and ALEs synthesis is realized through condensation reactions between electrophile and nucleophile reactants [17,21,22,23,24,25]. AGEs and ALEs are immunogen and they determine antibodies synthesis. AGEs initiate signaling cascades by specific receptors named RAGE. No data could be found in the literature about a receptor-mediated mechanism regarding the destructive effect of ALEs [21]. RAGE polymorphisms were associated with SLE susceptibility and lupus nephritis [26]. sRAGE could exert benefic effects by preventing proinflammatory signaling, they act as bait receiver [25,27,28,29,30,31]. 

In systemic autoimmune diseases, ROS and RNS overproduction induce DNA integrity alteration, damage of DNA response and repair (DDR/R) mechanisms, accumulation of mono- and double catenary cytosolic DNA, activation of stimulator of interferon genes (STING), synthesis of type 1 interferon [32]. DDR/R recognize defects during cell cycle and assures their correct reparation. In case of unrepaired lesions, the cell transmits the mutant genome to its descendants, otherwise it is neutralized by apoptosis or senescence [3,32,33].

The most frequent oxidative lesion in aerobe organisms is the formation of 7,8-dihydro-8-oxo-2′-deoxyguanosin (8-oxo-dG or 8-OH dG) [17,34,35]. In normal cells, during DNA replication, 2-deoxyguaninae (dG) is associated with 2′-deoxycitosine (dC). In tissues with high levels of oxidative stress, dG could wrongly link 2′-deoxyadenine (dA) and could induce G → C at T → A transversion. If 8-OHdG is not efficiently eliminated, it accumulates in tissue and induces genomic instability and cells dysfunctions [17,32,33,35]. Usually, 8-oxoguanine-DNA-glycosylase (OGG1) is responsible for 8-OH-dG clean-up. OGG1 deficit induces high levels of 8-OH-dG in DNA. OGG1 overexpression in mitochondria improves its function, cell survival and reduces the number of DNA lesions by 8-OH-dG reparation in oxidative stress conditions, in vitro. These data suggest that OGG1 could play a protective role in inflammatory diseases. OGG1 polymorphism could offer susceptibility to lupus nephritis and modulate 8-OH-d G serum level in SLE patients [36,37,38].

Carbonylated proteins, nitrotyrosine and oxidated glutathione are stable chemical products and they are used as biomarkers in SLE [16]. Increased levels of nitrates, nitrites, homocysteine and oxidated serum proteins are associated with tissue lesions and SLE activity. High SLEDAI was correlated with low serum albumin in lupus nephritis [15]. Some studies in the last few years showed that thiol-disulfides interconversion plays a crucial role in antioxidant defense, apoptosis, detoxification, transcription, enzymatic activity regulation [20,37,38].

All these data suggest that SLE patients have high risk of developing oxidative stress-associated inflammatory response. The effects of pharmacological therapies on oxidative stress depend on chemical characteristics of reactive metabolites and action mechanisms consequences. Based on these data, the present study tries to determine a pattern of oxidative stress markers and endogenous strategies for suppression/modulating oxidative stress in SLE patients. The aim of the study was to detect deficiencies in protective system of cells in order to minimize the consequences of oxidative stress and to identify individualized pharmacological targets in SLE patients.

## 2. Results

### 2.1. Clinical Characteristics of the Studied Groups

The paraclinical characteristics of the studied groups are presented in Table 1. Classic biomarkers of lupus activity such as anti-ds DNA, UACR, C1q, C3 and C4 complement proteins were assessed. dsDNA was statistically significantly higher in LN and non-LN groups when compared with control group (*p* ˂ 0.05), but it did not vary between SLE groups (*p* > 0.05). Urinary albumin: creatinine ratio was statistically significantly higher in LN group than in non-LN group (*p* ˂ 0.05) or in control group (*p* ˂ 0.05). C1q, C3 and C4 complement proteins were statistically significantly higher in LN group than in non-LN group (*p* ˂ 0.05) or in control group (*p* ˂ 0.05). Leucocytes and Hemoglobin were statistically significantly lower in SLE groups compared with the control group (*p* ˂ 0.05), but without statistical variation between LN and non-LN subjects (*p* > 0.05). Albumin was statistically significantly lower in SLE groups, when compared with control (*p* ˂ 0.05) and also in LN compared with non-LN group (*p* ˂ 0.05). The estimated glomerular filtration rate was found to be lower in LN group than in non-LN group (*p* ˂ 0.05) or in control group (*p* ˂ 0.05). Renal tubular injury was evaluated by measuring the urinary levels of b2-microglobulin, that was found to be higher in LN group than in non-LN group (*p* ˂ 0.05) or in control group (*p* ˂ 0.05). Inflammation was assessed by determination of erythrocyte sedimentation rate and C reactive protein, and we found high inflammation in SLE groups compared with control (*p* ˂ 0.05), but no significant variation between SLE groups (*p* > 0.05).

The clinical manifestations of SLE in non-LN and LN groups and the associated comorbidities are presented in Table 2.

### 2.2. Lipid Peroxidation Pattern in the Studied Groups

We evaluated lipid peroxidation pattern by determining serum levels of 4-HNE (μg/mL), TBARs (μmol/L), MDA (ng/mL), F2-Isoprostan (pg/mL) and ImAnOx (μmol/L). 4-HNE levels had an increase with 28.9% in non-LN group (*p* > 0.05) and with 43.22% in LN group (*p* ˂ 0.05), when compared with control group. TBARs levels were statistically significantly higher with 52.76% in non-LN group (*p* ˂ 0.05) and with 71.35% in LN group (*p* ˂ 0.01), when compared with control group. MDA increased with 93.3% in non-LN group (*p* ˂ 0.05) and with 73.9% in LN group (*p* ˂ 0.05) compared with control group. F2-Isoprostan also increased with 240% in LN group (*p* ˂ 0.01) and with 334% in non-LN group (*p* ˂ 0.01) compared with control group (*p* ˂ 0.01). ImAnOx decreased with statistical significance with 33.6% in LN group (*p* ˂ 0.01), and 14.3% in non-LN group (*p* ˂ 0.01) compared with control group. When comparing LN and non-LN groups, we detected a statistically significant increase in F2-Isoprostan and TBARS and decrease in ImAnOx in LN group (*p* ˂ 0.05). 4-HNE, TBARs and F2-Isoprostan had statistically significantly higher levels, while ImAnOx had statistically significantly lower levels in type IV lupus nephritis patients, when compared with SLE non-LN control group. When comparing SLE–LN patients with type IV lupus nephritis patients, we did not detect any statistically significant variations. All results are presented in Table 3. 

### 2.3. DNA Oxidation in Studied Groups

Oxidative DNA damage was evaluated by serum levels of 8-OHdG (ng/mL) and OGG1 status. 8-OHdG increased 1.16-fold in non-LN group (*p* ˂ 0.01), 1.17-fold in LN group (*p* ˂ 0.01), respectively 1.16 in type IV lupus nephritis (*p* < 0.05) compared with control group. There were no statistically significant variations between LN, type IV nephritis subjects and non-LN group. OGG1 did not vary significantly between non-LN and the control group, but in LN group it decreased 1.23-fold when compared with the control group (*p* < 0.001) and 1.28-fold when compared with non-LN group (*p* < 0.001). OGG1 did not vary significantly between SLE–LN and type IV LN subjects. All results are presented in Table 4.

### 2.4. Carbohydrate Oxidation in Studied Groups

Carbohydrate oxidation status was evaluated by assessment of serum pentosidine (ng/mL) and AGE (ng/mL)- sRAGE (pg/mL) axis. Pentosidine levels were 249% higher in non-LN group (*p* ˂ 0.01), 276% higher in LN group (*p* ˂ 0.01), and 278% higher in the type IV nephritis cohort (*p* ˂ 0.01) when compared with the control group. Pentosidine did not vary significantly between LN, non-LN group and type IV nephritis groups. AGE levels were 216% higher in non-LN group (*p* ˂ 0.01), 243% in LN group (*p* ˂ 0.01), and 267% in type IV nephritis (*p* ˂ 0.01), when compared with control group. sRAGE decreased with 7.6% in non-LN group (*p* ˂ 0.001), with 5.8% in LN group (*p* ˂ 0.001), with 5.5% in type IV nephritis (*p* ˂ 0.001), when compared with the control group. Pentosidine, AGE and sRAGE vary insignificantly both between LN–Non-LN group, and LN–Type IV nephritis group. All results are presented in Table 5.

### 2.5. Oxidized Protein Pattern in the Studied Groups

Protein oxidation was evaluated by serum levels of 3-nitrotyrosine (μmol/L), carbonylated proteins (PCO-μmol/L), native thiols (NT-μmol/L), total thiols (TT-μmol/L), disulphides (DS-μmol/L) and DS/NT, DS/TT and NT/TT ratios. Nitrotyrosine increased 2.23-fold in non-LN group (*p* < 0.001), and 3-fold in LN group (*p* < 0.001) when compared with control group. Nitrotyrosine varied statistically significantly between LN and non-LN group (*p* < 0.001). PCO increased 1.54-fold in non-LN group (*p* < 0.01), and 1.67-fold in LN group (*p* < 0.01) when compared with control group. PCO also varied statistically significantly between LN and non-LN group (*p* < 0.01). NT decreased by 1.12-fold in non-LN group (*p* < 0.001), and 1.23-fold in LN group (*p* < 0.001) when compared with the control group. NT varied statistically significantly between LN and non-LN group (*p* < 0.001). TT decreased 1-fold in non-LN group (*p* < 0.001), and 1.13-fold in LN group (*p* < 0.001) when compared with the control group. TT varied statistically significantly between LN and non-LN group (*p* < 0.001). DS increased 1.31-fold in non-LN group (*p* < 0.001), and 1.63-fold in LN group (*p* < 0.001) when compared with control group. DS varied statistically significantly between LN and non-LN group (*p* < 0.001). DS/NT increased 1.48-fold in non-LN group (*p* < 0.001), and 2.02-fold in LN group (*p* < 0.001) when compared with control group. DS/NT varied statistically significantly between LN and non-LN group (*p* < 0.001). DS/TT increased 1.58-fold in non-LN group (*p* < 0.001), and 2.06-fold in LN group (*p* < 0.001) when compared with the control group. DS/TT varied statistically significantly between LN and non-LN group (*p* < 0.001). NT/TT decreased 1.04-fold in non-LN group (*p* < 0.001), and 1.09-fold in LN group (*p* < 0.001) when compared with the control group. NT/TT varied statistically significantly between LN and non-LN group (*p* < 0.001). 3-NT, PCO, NT, TT, DS and DS/NT, DS/TT and NT/TT ratios had similar statistical variations when compared type IV lupus nephritis with SLE and controls and no statistical variations when compared with SLE–LN. All results are presented in Table 6.

### 2.6. Correlation Analysis between the Different Parameters Studied in SLE Patients

The correlation between some parameters were studied both in SLE patients, and LN-SLE patients by Pearson coefficient. Correlations of different parameters in SLE patients are presented in Table 7 and Table 8. We detected a negative, statistically significant correlation between ImAnOx and 4-HNE, TBARs, MDA, 8-oxo-dG and PCO. OGG-1 correlated negatively with statistical significance with 8-oxo-dG. NT and TT correlated negatively with statistical significance with 4-HNE, nitrotyrosine and PCO. DS correlated positively with statistical significance with TBARs, AGE, nitrotyrosine and PCO. DS/NT correlated positively, while DS/TT corelated negatively with nitrotyrosine and PCO. NT/TT negatively correlated with pentosidine, nitrotyrosine and PCO. sRAGE correlated negatively with pentosidine and AGE. Correlations of different parameters in LN patients are presented in Table 6. ImAnOx and 4-HNE, TBARs, MDA, F2Isoprostan, 8-oxo-dG and PCO. OGG-1 correlated negatively with statistical significance with 8-oxo-dG. NT and TT correlated negatively with statistical significance with 4-HNE, nitrotyrosine and PCO. DS correlated positively with statistical significance with TBARs, F2Isoprostan, 8-oxo-dG, pentosidine, AGE, nitrotyrosine and PCO. DS/NT correlated negatively with nitrotyrosine and PCOm while DS/TT corelated negatively only with PCO. NT/TT negatively correlated with pentosidine and PCO. sRAGE correlated negatively with pentosidine and AGE.

## 3. Discussion

The results of the present study showed that SLE patients have a high risk of inflammatory responses associated with oxidative stress. The assessment of oxidized metabolites pattern and endogenous strategies for suppression or modulating oxidative stress in SLE patients with or without renal impairment could be relevant in differential regulation of cellular response for preventing the progression of the renal manifestations. We determined a large panel of oxidative damage serum metabolites and endogenous protective mechanisms in SLE patients and a control group. The comparative analysis of oxidized metabolites pattern and antioxidative capacity in SLE with all type lupus nephritis, in type IV lupus nephritis, and in SLE without renal impairment aimed to determine possible deficiencies in protective system of the cells and some humoral biomarkers for detection of renal impairment in SLE patients.

The comparative analysis of lipoperoxidation process by simultaneous determination of 4-HNE, TBARs, MDA and F2-isoprostan in SLE patients and controls demonstrated higher levels of lipoperoxidation-derived reactive carbonyl species in SLE and LN compared with the control group. LN patients have statistically significantly higher levels of TBARs and F2-isoprostan, comparative with SLE non-LN patients. These major oxidative degradations were accompanied by low levels of ImAnOx in SLE patients versus controls. Serum antioxidants inactivation was more accentuated in LN patients compared with non-LN patients. ImAnOx capacity of blocking lipoperoxides formation or blocking lipids peroxidation propagation was demonstrated in this paper by negative association between lipid metabolites levels and antioxidant capacity of serum. Based on these results, we could appreciate that ImAnOx diminishing and F2-isoprostan increase are associated with LN development. As a parameter of oxidative deterioration, F2-isoprostan is considered by some publications as a useful oxidative stress biomarker [39,40]. F2 isoprostan varies according to pathologies; it has very low levels in cancer or cardiovascular diseases, very high in respiratory diseases and uro-genital disorders, or its value varies with clinical features [40] or inflammation, such as in SLE [39]. F2-isoprostan does not present specificity for oxidative stress due to the synthesis mechanisms. In our study, serum variability of F2-isoprostan in SLE and LN was caused by the level of oxidative stress, both SLE groups being characterized by a low inflammatory state. F2-isoprostan is useful in identifying patients at risk of developing LN.

Some studies show that lipid peroxidation plays a major role in SLE pathogenesis. SLE patients had higher levels of MDA and 8-OHdG and lower levels of TAS compared with controls. Circulant levels of MDA, 8-OhDG and TAS in patients with SLEDAI over six were statistically significant modified comparative with patients with SLEDAI below six. MDA presented a positive correlation and TAS a negative correlation with SLEDAI. Remarkable also is the high MDA/TAS ratio, in patients with neuro-psychiatric manifestations and vasculitis in SLE, high levels of oxidized low density lipoprotein cholesterol in SLE patients with thrombocytopenia and vasculitis [41]. Increases of MDA and 8-OhdG, and decreases of TAS were associated with disease activity. ROS role in SLE was evaluated by aldehydes specific immune complexes derived from lipids. These data sustain a possible relation between oxidative stress, anti-species carbonyl reactive, pathogenic mechanisms and prognosis in SLE [42]. A recent study evaluated oxidative stress markers, inflammation and biomarkers of activity in SLE and detected significant differences between active and inactive lupus nephritis for MDA, total and oxidated GSH, TAS, CRP, MCP 1, beta 2-microglobulin, urinary protein/creatinine ratio, dsDNA antibodies, anti-C1q antibodies and C3, C4. These data show a redox disequilibrium in patients with lupus nephritis determined by lipid peroxidation, process that affects glomerular basal membrane integrity and renal tubular function in these patients [43].

An interesting finding of our study, relevant for oxidative stress in SLE was represented by elevated oxidative DNA damage levels and defective DNA repair mechanisms via OGG1. Oxidative DNA lesions were overexpressed both in non-LN and LN groups comparative to controls. OGG1 has the ability to catalyze lesions excision of 8-oxo-dG type and assure genomic integrity in SLE patients. OGG1 capacity of DNA repair and of 8-oxo-dG serum levels modulating in LN patients is highly altered. Based on these results, we consider that 8-oxo-dG/OGG1 ratio could offer information about LN development risk. DNA alteration by oxidative stimuli, DNA repair mechanisms dysfunctions and immune response alteration in cellular defense were intensively studied in the last years [3]. DNA alteration could induce aberrant activation of innate immunity, and, associated with chronic inflammation, could stimulate DDR/R network. ROS and RNS generated by inflammatory cells promote 8-oxo-dG and 8-nitro-dG overexpression resulting in the alteration of the repair mechanisms of DNA lesions. Still, in SLE, there is proved a bidirectional interaction between DDR/R and immune response induced by oxidative stress. DDR/R network and immune innate response act synergistically for the survival of all living organisms. Epigenetically regulated functional abnormalities of DNA repair mechanisms could lead to increased accumulation of DNA lesions. This accumulation facilitates the production of autoantibodies, the generation of damaged cytosolic DNA and micronuclei that can act as powerful stimulators of the immune system by over-expression of the cGAS-STING-IRF3 path and the production of type I IFN, leading to the systemic expression of autoimmune diseases [3,14,32].

Rigorous research of carbohydrate oxidation in this study showed profound alteration of the AGE–RAGE axis in patients with SLE and LN. The harmful effect of AGE on human tissues was proved by excessive production of RAGE ligands in the studied patients. Thus, serum levels of AGE and pentosidine were significantly increased in patients with SLE and LN compared to control. The toxic action of AGE was limited by sRAGE. The protective effect of sRAGE was well expressed in patients with SLE. On the other hand, the ability of sRAGE to block activation of RAGE in patients with LN was affected. The different behavior of sRAGE in SLE with or without LN could be sustained by negative correlation between AGE, and pentosidine and sRAGE in non-LN patients and their positive association in LN patients. It can be interpreted that an increased amount of AGE in patients’ blood leads to increased consumption of sRAGE. These findings support the quality of sRAGE as a biomarker to differentiate patients with SLE at risk of kidney injury. Recent findings suggest that the occurrence of SLE depends on the interactions between genetic background and risk factors, such as exposure to oxidative stimuli. Chronic inflammation associated with SLE disturbs the oxidant/antioxidant balance. These processes are related to the excess accumulation of AGE. Lately, the accumulation of AGE in the skin in patients with autoimmune diseases and the expression of corresponding receptors gain the attention of researchers. These AGE compounds could be found in inflamed tissues. The reference data present in the literature show a link between oxidative stress, AGE formation and autoimmune diseases such as SLE [44,45]. The most representative AGEs are pentosidine, carboxymethyllysine (CML) and carboxiethyllysine (CEL). The high levels of AGEs in patients’ blood are still being discussed. Some authors indicated that there were no significant differences between SLE patients and controls regarding plasma levels of CML and CEL. AGE levels did not correlate with CRP. The authors suggested that AGE accumulated mainly in tissues and plasma proteins [45,46]. Additionally, the level of pentosidine did not increase in the blood of patients with SLE. Some patients with increased fructosamine showed remarkably high levels of pentosidine. Other authors demonstrated an increase in plasma AGE in patients with SLE. In addition, the levels of CML and pentosidine showed a positive correlation with the SLEDAI score. 

Other RAGE ligands, such as S100A12 levels are similar in SLE and healthy subjects, while levels of S100A8/A9 and S100A12 correlate with cardiovascular risk in SLE patients [47,48,49]. RAGE ligands appear to be mostly related to inflammation, hyperglycation and cellular stress processes; expression of the receptor itself being directly related to inflammation [50,51]. 

sRAGE can bind to RAGE, blocking its functions and leading to an improved expression of the molecules involved in inflammation, adhesion and RAGE itself [47].

RAGE expression is also enhanced by TNF-α. It remains unknown whether patients with SLE have autoantibodies against RAGE/sRAGE, but sRAGE functions are insufficient studied in this pathology [9]. A low amount of sRAGE has been detected in Sjogren’s syndrome, though a possible link between SLE and visual disturbances could be established [47]. Contradictory data on the level of sRAGE have been reported in SLE patients. Some authors have reported increased concentrations of sRAGE in the serum of lupus patients [45]. Additionally, sRAGE positively correlates with SLEDAI [52]. Other studies showed the decreased levels of sRAGE in the plasma of SLE patients compared to control. Notably, SLE patients treated or untreated have similar levels of sRAGE, while subjects who received long-term treatment have a higher concentration of soluble receptor than subjects receiving short-term one. Short-term treatment induces a rapid decrease in sRAGE levels compared to long-term one. It is suggested that the receptor has a different role in the initial and progressive stage of the disease [47,48]. In addition, patients with SLE who have antiphospholipid antibodies (APA) or antiphospholipid syndrome (APS) have shown a decrease in sRAGE plasma levels compared to control [53]. sRAGE level is associated with leukocytes lymphocytes, neutrophils and monocytes number and with C4 levels, but it does not correlate with the presence of autoantibodies. sRAGE therefore participates in the development of inflammation and the recruitment of leukocytes. RAGE and sRAGE have the ability to interact with each other and with multiple ligands, and they are directly related to the immune. These processes increase the oxidative stress in the body. AGE and RAGE with sRAGE deficiency can cause the formation of neoepitopes, and of autoantibodies [47].

Finally, we found that changes in oxidative proteins and TDH regulators play a central role in the cellular response to oxidative stress. In this paper, it was found that 3-nitrotyrosine, carbonylated proteins and disulfides were overexpressed in patients with SLE and LN compared to the healthy population. The circulating level of 3-nitrothyrosine was significantly increased in patients with LN versus patients with SLE non-LN. The ability of TDH to maintain redox homeostasis of cells has also been demonstrated by the inverse association between the concentrations of sulfhydryl groups and oxidized proteins in both SLE and LN. Through their dynamics, the DS/TN, DS/TT, TN/TT ratios can be potential serum biomarkers that have managed to differentiate between SLE patients, those that will develop LN. Our findings suggest that protein oxidation may play a substantial role in the pathogenesis of chronic kidney damage in patients with SLE. These results reconfirm data from previously published studies regarding levels of multiple markers of protein oxidation. SOD and myeloperoxidase activities were increased, while thiol protein levels, glutathione peroxidase and catalase activities were reduced in serum of SLE patients compared to controls. Disease activity markers were positively correlated with erythrocyte sedimentation rate and carbonylated protein levels, 3-nitrosyrosine and CRP, and negatively correlated with protein thiol levels and SOD, glutathione peroxidase and catalase activities in patients with SLE. There were significant differences in serum levels of carbonylated proteins between patients with and without kidney disease [54]. 

Lipid peroxides (LOOH), advanced oxidation protein products (AOPP), nitrogen oxides (NOx), sulfhydryl groups (−SH), oxidative degradation products of deoxyribonucleic acid (DNA)/ribonucleic acid (RNA) and antioxidant parameter of radicals’ uptake (TRAP) participates in SLE immune pathophysiology and modulates disease severity and adhesion molecule expression [55,56,57]. Another study showed that increased protein oxidation correlated with SLEDAI score and dsDNA levels [58]. Other publications have shown that oxidative stress is involved in the pathogenesis of LN. Higher levels of serum AOPP have been associated with an increased risk of LN. In contrast, neither high levels of dsDNA nor low levels of C3 were independent risk factors for LN [59]. Evaluation of the prooxidant–antioxidant balance (PAB) showed increased values in patients with SLE with alopecia, discoid rash, oral ulcers, arthritis and nephritis. These findings suggest that PAB measurement may be useful to show the state of oxidative stress in patients with SLE [60].

To resume, the present study is the first one in the literature that presents a pattern of oxidative stress markers and endogenous strategies for suppression/modulating oxidative stress in SLE patients. However, some limitations should be noted. Our study followed patients with SLE non-LN and LN, for a period of three years, only with chronic immunosuppressant treatment. Further studies with a larger number of patients with different therapeutic regimens should be developed. Most of the patients included in the study had type IV nephritis. For a better evaluation of oxidant/antioxidant axis in SLE a larger number of patients with all types of nephritis are needed, although it would be very hard to identify these patients because they have minimal symptoms. In LN patients, it is very important to identify as early as possible the patients at risk for LN development, in order to avoid invasive interventions and to establish an effective medical intervention.

## 4. Materials and Methods

### 4.1. Study Participants

The present study is prospective–observational and included 82 SLE patients and 40 healthy subjects. All the patients signed the informed consent, the Declaration of Helsinki from 1975 was respected. The study was developed between 2018 and 2021, and patients over 18 years old were selected from those who attended the Clinical Hospital of Nephrology “Carol Davila” and Clinical Hospital “Victor Babes”. The study protocol was approved by the Ethics Committee of Clinical Hospital of Nephrology “Carol Davila” (11/23.07.2018). Of these 82 SLE patients, 44 had SLE with cutaneous and hematological determinations, but no lupus nephritis (non-LN group), while 38 had lupus nephritis (LN group) diagnosed by biopsy puncture and histological exam according to KDIGO guidelines. The activity and chronicity index of lupus nephritis was evaluated and it is presented in Table 9. In LN patients, 7% had type II LN, 18% had type III LN, 70% had type IV and 5% type V. SLE diagnosis was established according to Systemic Lupus International Collaborating Clinics/American College of Rheumatology criteria. The activity disease was based on clinical Systemic Lupus Erythematosus Disease Activity Index (SLEDAI). The time lapse of disease and ongoing treatment (non-steroidal anti-inflammatory drugs, corticosteroids, hydroxychlorochine, immunosuppressant drugs such as azathioprine, mycophenolate mophetil, antihypertensive therapy) were recorded for each patient. The exclusion criteria were the presence of any cardiovascular, hepatic, thyroid, gastrointestinal, or oncological disease, any viral or bacterial infections in the last three months, tobacco use, drug abuse, alcoholism, use of vitamin or other antioxidant supplements, and pregnancy.

### 4.2. Laboratory Data

The blood samples were collected from all the study participants, after signing the informed consent, who fasted 12 h, using a holder-vacutainer system and ×3000 g, for ten minutes, after one hour of keeping at room temperature. The sera were separated and frozen at −80 degrees before analyzing. 

4-HNE and MDA were assessed by competitive ELISA method (semi-automatic Tecan analyzer). The wells were pre-coated with substrate and the final product colorimetric evaluation was made at 450 nm. The results were expressed as microgram/mL serum for 4-HNE and as nanogram/mL serum for MDA. MDA forms a complex with thiobarbituric acid reactive substances (TBARS) that is measured using the spectrophotometric method (BS-3000M Semi-Automatic Chemistry Analyzer) and read at a wave-length of 532 nm. The results were expressed as μmol/L serum. 8-Isoprostan was determined by competitive ELISA method (semi-automatic Tecan analyzer). The wells were pre-coated with substrate to acetylcholinesterase and the final product colorimetric evaluation was made at 412 nm. Results were expressed in pg/mL. ImAnOx was assessed by spectrophotometry (semi-automatic Tecan analyzer). by the reaction of antioxidants in the sample with a defined amount of exogenously provided hydrogen peroxide. The antioxidants in the sample eliminate a certain amount of the provided hydrogen peroxide. The residual H2 O2 is determined photometrically at 450 nm. Results were expressed in μmol/L.

8-OHdG was determined by competitive ELISA method (semi-automatic Tecan analyzer). The wells were pre-coated with a target specific capture antibody and the final product colorimetric evaluation was made at 450 nm. Results were expressed in ng/mL. OGG1 was determined by ELISA method, quantitative sandwich (semi-automatic Tecan analyzer). The wells were pre-coated with a target specific capture antibody and the final product colorimetric evaluation was made at 450 nm. Results were expressed in pg/mL.

Pentosidine and AGEs were assessed by ELISA method (semi-automatic Tecan analyzer). The wells were pre-coated with a target specific capture antibody and the final product colorimetric evaluation was made at 450 nm. Results were expressed in ng/mL. sRAGE was determined using the sandwich ELISA method (immunoenzymatic kits-R&D SYSTEMS (DR600 and SRG00 kits), USA), the results were read at 450 nm, using a TECAN analyzer (Tecan, Switzerland). The sensitivity of the method was 16.14 pg/mL and assay range was between 78 and 5000 pg/mL.

Circulant 3-NT was determined by ELISA method using the R&D systems reactive (MAB3248 kit) and a TECAN analyzer (Tecan, Switzerland).

Carbonyl groups were assessed by spectrophotometric methods, in reaction with 2,4-dinythrophenylhydrasine that generated hydrazone, employing the HumanStar300 analyzer (HUMAN Gesellschaft für Biochemica und Diagnostica mbH, Weisbaden, Germany) and Merck reactives (MAK094 kit).

Thiol disulphide homeostasis parameters (TDHPs) were determined using a spectrophotometric method The dynamic and reducible disulfide bonds were transformed into free functional thiol groups by using sodium borohydride (NaBH4, 10 mM) as follows:

R-S2-R ‘+ NaBH4 → 2 R-SH + BH3 + Na.

Subsequently, the amount of NaBH4, which have not participated in the reaction, was removed with formaldehyde (10 mM, pH 8.2). The levels of native thiol (TN) and total thiol (TT) were assessed using 5,5′-dithiobis-2-nitrobenzoic acid (DTNB, 10 mM) as follows:

R-SH + DTNB → R-TNB + TNB.

The final product, 2-nitro-5-thiobenzoate (TNB), ionized at alkaline pH and turned yellow. An automatic biochemistry analyzer (HumaSTAR 300, (HUMAN Gesellschaft für Biochemica und Diagnostica mbH, Weisbaden, Germany) and Merck reactives were used. This technique allows for the assessment of functional disulfide bonds in the sample. Disulfide (DS) level was calculated as half of the difference between TT and NT. The levels of TT, NT and DS were expressed as μmol/L serum. The disulfide/native thiol ratio (DS/NT), disulfide/total thiol ratio (DS/TT), and native thiol/total thiol ratio (NT/TT) were calculated and expressed as %. TDHPs were represented by:

- NT (-SH), determined by a spectrophotometric method;
- TT (-SH + -S-S-), determined by a spectrophotometric method;
- DS (-S-S), determined by calculation;
- DS/NT (-S-S- ∗ 100/-SH) was calculated;
- DS/TT (-S-S- ∗ 100/-SH + -S-S-) was calculated;
- NT/TT (-SH ∗ 100/-SH + -S-S-) was calculated.

### 4.3. Statistical Analysis

We used mean and standard deviation for data presentation. We compared the data using either the analysis of variance (ANOVA) with Tukey post hoc test or Kruskal–Wallis test with Dunn’s post hoc test for normally and non-normally distributed data. The relation between the studied parameters was assessed by Pearson’s correlation coefficient, but before the assessment, data normality by the Kolmogorov–Smirnov test was evaluated. The level of significance (p) chosen was 0.05 (5%) and the confidence interval was 95% for hypothesis testing and the corresponding ethical approval code.

## 5. Conclusions

SLE is a chronic autoimmune disease with complex pathogenesis, characterized by oxidative stress and high inflammatory state. The cellular response to oxidative stimuli in this pathology is concreted in the amplification of oxidative degradation of lipids, proteins, nucleic acid, and hydro carbonates, and in alteration of endogenous strategies for suppressing/modulating oxidative stress. The defective DNA repair mechanism via OGG1, the reduced regulatory effect of sRAGE in the activation of AGE-RAGE axis, low levels of thiols, disulphide bonds formation and high nitrotyrosination are important characteristics of lupus nephritis in our study and they could explain alteration of renal architecture and development of renal injury in SLE. All these data help us to establish a panel of biomarkers in order to identify as early as possible the patients at risk for LN development, thus avoiding invasive interventions such as renal biopsy, and establishing an effective treatment as soon as possible from diagnosis. The identification of more molecular mechanisms to counteract oxidative stress in LN could permit a more precise assessment of disease prognosis, as well as the development of new therapeutic targets.

## Figures and Tables

**Table 1:** Patients’ characteristics.

Characteristics	SLE–Non-LN	LN	Control	*p* Significance
Number of patients	44	38	40	0.47
Women:Men ratio	2/1	2/1	2/1	0.89
Age (years)	42.6 ± 6.3	44.8 ± 5.5	43.7 ± 6.2	0.56
Disease duration (years)	6.4 ± 1.3	6.7 ± 1.1	−	0.28
SLEDAI	6.2 ± 4.7	7.4 ± 5.3	−	0.35
dsDNA (IU/mL)	320.1 ± 122.4	342.7 ± 98.2	80.2 ± 16.2	**0.02**
BMI (Kg/mp)	22.9 ± 1.7	23.0 ± 2.1	22.0 ± 3.3	0.64
Leucocytes (cells/mmc)	3800 ± 2008	4050 ± 1040	5860 ± 1070	**0.03**
Haemoglobin (g/L)	10.3 ± 1.1	10.8 ±0.8	12.8 ± 1.2	**0.02**
Phosphorus (mg/dL)	3.6 ± 0.7	3.6 ± 0.9	3.7 ± 0.6	0.41
Calcium (mg/dL)	9.11 ± 0.4	9.18 ± 0.6	9.22 ± 0.57	0.28
LDH (U/L)	297 ± 65	307 ± 63	316 ± 57	0.08
Glycemia (mg/dL)	84.1 ± 11.3	78.7 ± 13.7	82.7 ± 11.4	0.11
ASAT (U/L)	19.4 ± 11.2	17.2 ± 8.4	19.2 ± 8.7	0.24
ALAT (U/L)	17.1 ± 13.2	16.2 ± 6.2	20.2 ± 10.3	0.08
Cholesterol (mg/dL)	152.5 ± 21.2	146.2 ± 24.1	142.5 ± 19.9	0.07
Triglycerides (mg/dL)	89.5 ± 12.7	81.4 ± 10.5	86.7 ± 15.5	0.23
Albumin (g/dL)	3.26 ± 0.25	3.68 ± 0.34	4.01 ± 0.44	**<0.04**
Urea (mg/dL)	35.4 ± 11.4	34.6 ± 0.7	30.2 ± 0.9	0.08
Creatinine (mg/dL)	1.0 ± 0.22	1.28 ± 0.11	0.77 ± 0.12	**0.02**
eGFR (mL/min/1.73 mp)	91.45 ± 12.23	82.17 ± 17.12	97.82 ± 7.22	**0.02**
Uric acid (mg/dL)	4.2 ± 1.3	3.8 ± 1.0	4.2 ± 1.2	0.13
UACR (mg/g creatinine)	10.81 ± 3.88	19.84 ± 2.97	7.34 ± 0.22	**0.03**
Haematuria (sw-RBC/camp)	5 ± 4	16 ± 7	3 ± 1	**0.02**
Leucocyturia (sw-leuc/camp)	5.3 ± 1.1	6.8 ± 0.9	3.8 ± 2.7	0.07
Urinary b2-microglobulin (mg/L)	0.22 ± 0.07	0.38 ± 0.14	0.12 ± 0.04	**0.04**
ESR (mm/h)	21.7 ± 17.1	24.5 ± 11.0	5.7 ± 5.0	**0.02**
CRP (mg/dL)	1.04 ± 0.72	1.29 ± 0.35	0.14 ± 0.12	**0.02**
C3 (mg/dL)	82.4 ± 21.2	70.5 ± 15.8	98.5 ± 25.1	**0.03**
C4 (mg/dL)	10.2 ± 1.7	7.4 ± 1.3	19.4 ± 9.3	**0.01**
C1q (mg/dL)	4.22 ± 0.95	3.02 ± 1.1	15.4 ± 6.3	**0.04**

SLEDAI—Systemic Lupus Erythematosus Disease Activity Index, BMI—body mass index; LDH—lactatdehydrogenase; ASAT—aspartate aminotransferase; ALAT—alanyl aminotransferase; eGFR—estimated glomerular filtration rate; UACR—urinary albumin: creatinine ratio; ESR—erythrocyte sedimentation rate; CRP—C reactive protein; *p*—statistical significance.

**Table 2:** Clinical characteristics of the SLE groups.

Clinical Characteristics	SLE—Non-LN	LN	*p* Significance
Number of patients	44	38	0.47
Constitutional symptoms (fatigue, fever, weight loss)	40	33	0.07
Mucocutaneous (malar rash, alopecia, mucosal ulcers, discoid lesions)	41	3	**0.02**
Musculoskeletal (arthritis/arthralgia, myositis, avascular necrosis)	21	10	0.05
Neuropsychiatric involvement (depression, seizures, demyelinating syndromes, peripheral neuropathy)	3	1	0.26
Pulmonary manifestations (pleural effusion)	1	−	0.68
Cardiac manifestation (pericarditis)	1	−	0.68
**Co-morbidities**			
Arterial Hypertension	14	29	**0.04**
Heart failure	4	1	0.08
History of myocardial infarction	2	−	0.16
Chronic pulmonary disease	3	1	0.21
Diabetes mellitus without organ damage	2	1	0.37
Hepatic steatosis	3	1	0.22
Osteoporosis	1	3	0.18

**Table 3:** Lipid peroxidation pattern in the studied group.

Metabolites	SLE–Non LN	SLE–LN	Type IV Lupus Nephritis	Control	*p*1	*p*2
4-HNE (µg/mL)	18.17 ± 4.02	20.18 ± 6.08	19.92 ± 5.88	14.09 ± 1.42	0.04	AB = 0.04
						AC = 0.04
						AD = 0.04
						BC = 0.07
						BD = 0.02
						CD = 0.02
TBARs (µmol/L)	3.04 ± 0.51	3.41 ± 0.66	3.39 ± 0.62	1.99 ± 0.14	0.03	AB = 0.04
						AC = 0.03
						AD = 0.006
						BC = 0.05
						BD = 0.003
						CD = 0.004
MDA (ng/mL)	38.02 ± 5.37	35.11 ± 5.94	36.12 ± 6.01	20.18 ± 1.22	0.04	AB = 0.08
						AC = 0.07
						AD = 0.02
						BC = 0.06
						BD = 0.04
						CD = 0.04
8-Isoprostan (pg/mL)	21.80 ± 6.14	27.87 ± 6.15	28.19 ± 5.88	6.41 ± 0.72	0.007	AB = 0.02
						AC = 0.03
						AD = 0.007
						BC = 0.05
						BD = 0.002
						CD = 0.003
ImAnOx (μmol/L)	259.3 ± 61.2	201.0 ± 77.4	197.12 ± 72.3	302.6 ± 13.1	0.0004	AB = 0.03
						AC = 0.04
						AD = 0.001
						BC = 0.04
						BD = 0.0005
						CD = 0.0004

4-HNE—4-hydroxy-2-nonenal, TBARs—Thiobarbituric acid reactive substances, MDA—Malondialdehyde, ImAnOX—Total antioxidative capacity, SLE—Systemic lupus erythematosus, LN—Lupus nephritis, *p*—Statistical significance, *p*1—Triple comparison of the groups, *p*2—Pairwise comparison of the groups, A—SLE non-LN, B—LN, C—type IV lupus nephritis, D—Control.

**Table 4:** DNA oxidation in studied groups.

Metabolite	SLE–Non LN	SLE–LN	Type IV Lupus Nephritis	Controls	*p*1	*p*2
8-OHdG (ng/mL)	3.58 ± 0.68	3.61 ± 0.81	3.59. ± 0.88	3.06 ± 0.43	0.007	AB = 0.08
						AC = 0.08
						AD = 0.005
						BC = 0.07
						BD = 0.005
						CD = 0.005
OGG1 (pg/mL)	20.80 ± 2.26	16.21 ± 3.31	16.32 ± 3.61	20.04 ± 1.07	0.0006	AB = 0.0007
						AC = 0.004
						AD = 0.0004
						BC = 0.09
						BD = 0.0006
						CD = 0.0005

8-OHdG—7,8-dihydro-8-oxo-2′-deoxyguanosine, OGG1—8-oxoguanine-DNA-glycosylase, SLE—Systemic lupus erythematosus, LN—Lupus nephritis, *p*—Statistical significance, *p*1—Triple comparison of the groups, *p*2—Pairwise comparison of the groups, A—SLE non-LN, B—LN, C—type IV lupus nephritis, D—Control.

**Table 5:** Carbohydrate oxidation in studied groups.

Metabolite	SLE–Non LN	SLE–LN	Type IV Lupus Nephritis	Controls	*p*1	*p*2
Pentosidine (ng/mL)	3.91 ± 0.79	4.22 ± 0.85	4.26 ± 0.88	1.12 ± 0.13	0.002	AB = 0.05
						AC = 0.06
						AD = 0.009
						BC = 0.18
						BD = 0.006
						CD = 0.005
AGE (ng/mL)	38.11 ± 4.61	41.38 ± 4.02	42.29 ± 4.62	12.04 ± 1.82	0.004	AB = 0.08
						AC = 0.06
						AD = 0.005
						BC = 0.05
						BD = 0.008
						CD = 0.007
sRAGE (pg/mL)	963.1 ± 164.3	982.4 ± 201.7	986.12 ± 206.01	1043 ± 123.1	0.0009	AB = 0.06
						AC = 0.05
						AD = 0.001
						BC = 0.07
						BD = 0.0008
						CD = 0.0004

AGE—Advanced glycation end products, sRAGE—Soluble receptor for advanced glycation end products—Systemic lupus erythematosus, LN—Lupus nephritis, *p*—Statistical significance, *p*1—Triple comparison of the groups, *p*2—Pairwise comparison of the groups, A—SLE non-LN, B—LN, C—type IV lupus nephritis, D—control.

**Table 6:** Oxidized protein pattern in the studied groups.

Metabolite	SLE–Non LN	SLE–LN	Type IV Lupus Nephritis	Controls	*p*1	*p*2
Nitrotyrosine (µmol/L)	0.29 ± 0.04	0.40 ± 0.11	0.39 ± 0.09	0.13 ± 0.02	0.0003	AB = 0.0003
						AC = 0.0004
						AD = 0.0004
						BC = 0.31
						BD = 0.0002
						CD = 0.0004
PCO (µmol/L)	34.82 ± 5.84	37.72 ± 6.01	38.79 ± 6.11	22.51 ± 2.21	0.007	AB = 0.009
						AC = 0.008
						AD = 0.005
						BC = 0.05
						BD = 0.004
						CD = 0.003
NT (µmol/L)	355.92 ± 8.53	324.21 ± 19.32	324.40 ± 18.81	401.83 ± 4.89	0.0008	AB = 0.005
						AC = 0.004
						AD = 0.0004
						BC = 0.09
						BD = 0.0007
						CD = 0.008
TT (µmol/L)	407.23 ± 7.40	388.12 ± 6.89	387.99 ± 6.42	440.89 ± 4.78	0.0004	AB = 0.0008
						AC = 0.0005
						AD = 0.0004
						BC = 0.09
						BD = 0.0002
						CD = 0.0002
DS (µmol/L)	25.65 ± 1.62	31.95 ± 2.97	31.40 ± 3.01	19.50 ± 0.53	0.0003	AB = 0.0006
						AC = 0.0004
						AD = 0.0004
						BC = 0.12
						BD = 0.0004
						CD = 0.0005
DS/NT	7.21 ± 0.55	9.84 ± 0.72	9.47 ± 0.56	4.85 ± 0.15	0.0008	AB = 0.0005
						AC = 0.0007
						AD = 0.0002
						BC = 0.22
						BD = 0.0003
						CD = 0.0003
DS/TT	6.30 ± 0.42	8.23 ± 0.76	8.34 ± 0.61	3.98 ± 1.27	0.0006	AB = 0.0007
						AC = 0.0005
						AD = 0.0004
						BC = 0.08
						BD = 0.0005
						CD = 0.0004
NT/TT	87.39 ± 0.85	83.35 ± 0.94	83.69 ± 0.72	91.15 ± 0.25	0.0003	AB = 0.0007
						AC = 0.0006
						AD = 0.0003
						BC = 0.07
						BD = 0.0002
						CD = 0.0004

PCO—Carbonylated proteins, NT—Native thiol, TT—Total thiol, DS—Disulphide, SLE—Systemic lupus erythematosus, LN—Lupus nephritis, *p*—Statistical significance, *p*1—Triple comparison of the groups, *p*2—Pairwise comparison of the groups, A—SLE non-LN, B—LN, C—type IV lupus nephritis, D—Control.

**Table 7:** Correlation analysis between studied parameters in SLE patients.

Metabolite	ImAnOx	OGG-1	NT	TT	DS	DS/NT	DS/TT	NT/TT	sRAGE
4-HNE	*r*= −0.42	NS	*r* = −0.43	*r* = −0.31	NS	NS	NS	NS	NS
	*p* = 0.02		*p* = 0.02	*p* = 0.04					
TBARs	*r* = −0.37	NS	NS	NS	*r* = 0.26	NS	NS	NS	NS
	*p* = 0.02				*p* = 0.05				
MDA	*r* = −0.23	NS	NS	NS	NS	NS	NS	NS	NS
	*p* = 0.03								
F2Isoprostan	NS	NS	NS	NS	NS	NS	NS	NS	NS
8-oxo-dG	*r* = −0.14	*r* = −0.26	NS	NS	NS	NS	NS	NS	NS
	*p* = 0.04	*p* = 0.04							
Pentosidine	NS	NS	NS	NS	NS	NS	NS	*r* = −0.34	*r* = −0.29
								*p* = 0.007	*p* = 0.03
AGE	NS	NS	NS	NS	*r* = 0.45	NS	NS	NS	*r* = −0.57
					*p* = 0.01				*p* = 0.007
Nitrotyrosine	NS	NS	*r* = −0.44	*r* = −0.26	*r* = 0.52	*r* = 0.53	*r* = −0.28	NS	NS
			*p* = 0.006	*p* = 0.008	*p* = 0.002	*p* = 0.01	*p* = 0.04		
PCO	*r* = −0.71	NS	*r* = −0.49	*r* = −0.63	*r* = 0.58	*r* = 0.32	*r* = −0.23	*r* = −0.21	NS
	*p* = 0.0006		*p* = 0.02	*p* = 0.006	*p* = 0.03	*p* = 0.04	*p* = 0.04	*p* = 0.04	

4-HNE—4-hydroxy-2-nonenal, TBARs—Thiobarbituric acid reactive substances, MDA—Malondialdehyde, ImAnOX—Total antioxidative capacity, 8-Oxo-dG—7,8-dihydro-8-oxo-2′-deoxyguanosin, OGG1—8-oxoguanine-DNA-glycosylase, AGE—Advanced glycation end products, sRAGE—Soluble receptor for advanced glycation end products, PCO—Carbonylated proteins, NT—Native thiol, TT—Total thiol, DS—Disulphide, SLE—Systemic lupus erythematosus, LN—Lupus nephritis, *p*—Statistical significance.

**Table 8:** Correlation analysis between studied parameters in LN patients.

Metabolite	ImAnOx	OGG-1	NT	TT	DS	DS/NT	DS/TT	NT/TT	sRAGE
4-HNE	*r* = −0.73	NS	*r* = −0.51	*r* = −0.38	NS	NS	NS	NS	NS
	*p* = 0.007		*p* = 0.02	*p* = 0.03					
TBARs	*r* = −0.62	NS		NS	*r* = 0.26	NS	NS	NS	NS
	*p* = 0.004				*p* = 0.05				
MDA	*r* = −0.45	NS	NS	NS	NS	NS	NS	NS	NS
	*p* = 0.004								
F2Isoprostan	*r* = −0.79	NS	NS	NS	*r* = 0.62	NS	NS	NS	NS
	*p* = 0.008				*p* = 0.01				
8-oxo-dG	*r* = −0.38	*r* = −0.88	NS	NS	r = 0.44	NS	NS	NS	NS
	*p* = 0.01	*p* = 0.0005			*p* = 0.01				
Pentosidine	NS	NS	NS	NS	*r* = 0.37	NS	NS	*r* = −0.28	*r* = 0.91
					*p* = 0.03			*p* = 0.04	*p* = 0.0007
AGE	NS	NS	NS	NS	*r* = 0.29	NS	NS	NS	*r* = 0.86
					*p* = 0.03				*p* = 0.0004
Nitrotyrosine	NS	NS	*r* = −0.39	*r* = −0.42	*r* = 0.73	*r* = −0.28	NS	NS	NS
			*p* = 0.004	*p* = 0.0006	*p* = 0.0003	*p* = 0.03			
PCO	*r* = −0.84	NS	*r* = −0.67	*r* = −0.73	*r* = 0.86	*r* = −0.12	*r* = −0.31	*r* = −0.29	NS
	*p* = 0.0005		*p* = 0.02	*p* = 0.004	*p* = 0.03	*p* = 0.04	*p* = 0.01	*p* = 0.04	

4-HNE—4-hydroxy-2-nonenal, TBARs—Thiobarbituric acid reactive substances, MDA—Malondialdehyde, ImAnOX—Total antioxidative capacity, 8-Oxo-dG—7,8-dihydro-8-oxo-2′-deoxyguanosin, OGG1—8-oxoguanine-DNA-glycosylase, AGE—Advanced glycation end products, sRAGE—Soluble receptor for advanced glycation end products, PCO—Carbonylated proteins, NT—Native thiol, TT—Total thiol, DS—Disulphide, SLE—Systemic lupus erythematosus, LN—Lupus nephritis, *p*—statistical significance.

**Table 9:** Activity and chronicity index in lupus nephritis.

NIH Activity Indices		Number of Patients
Endocapillary proliferation	0	−
	1	4
	2	15
	3	19
Glomerular leucocyte infiltration	0	1
	1	4
	2	12
	3	15
Hialin deposits/wire loops	0	9
	1	14
	2	10
	3	5
Fibrinoid necrosys (x2)	0	7
	1	11
	2	8
	3	9
Cellular of fibrocellular crescents (x2)	0	16
	1	10
	2	7
	3	5
Interstitial inflammation	0	-
	1	10
	2	16
	3	12
Chronicity indices		
Global glomerulosclerosys	0	21
	1	8
	2	5
	3	4
Fibrous crescents	0	27
	1	6
	2	3
	3	2
Tubular atrophy	0	24
	1	8
	2	4
	3	2
Interstitial fibrosis	0	15
	1	12
	2	6
	3	2
	4	3

## Data Availability

The data presented in this study are available on request from the corresponding author.
